# TNFA deletion alters apoptosis as well as caspase 3 and 4 expression during otitis media

**DOI:** 10.1186/1471-2172-12-12

**Published:** 2011-01-26

**Authors:** Joerg Ebmeyer, Anke Leichtle, Michelle Hernandez, Umay Ebmeyer, Jacob Husseman, Kwang Pak, Holger Sudhoff, David Broide, Stephen I Wasserman, Allen F Ryan

**Affiliations:** 1 Department of Otorhinolaryngology, Head and Neck Surgery Klinikum Bielefeld (Academic Teaching Hospital University of Münster), Bielefeld, Germany; 2 Department of Surgery, Division of Otolaryngology, University of California, San Diego School of Medicine and Department of Veterans Affairs Medical Center, La Jolla, California, USA; 3 Department of Medicine, Division of Rheumatology, Allergy and Immunology, UCSD School of Medicine, La Jolla, California, USA; 4 Department of Obstetrics and Gynecology Städtische Klinikum Bielefeld (Academic Teaching Hospital University of Münster), Bielefeld, Germany

## Abstract

**Background:**

Tumor necrosis factor (TNFA) is the canonical member of the TNF superfamily, which plays a major role in both inflammation and apoptosis. To evaluate the role of TNFs in otitis media (OM), the most common disease of childhood, we evaluated middle ear (ME) expression of genes encoding the TNF and TNF receptor superfamilies during bacterial OM in the mouse, characterized OM in TNFA-deficient mice, and assessed apoptosis during OM in normal versus TNF-deficient MEs.

**Results:**

TNFs and TNF receptors were broadly regulated during OM, with TNFA showing the highest level of up-regulation. TNF deficient mice exhibited mucosal hyperplasia even in the absence of infection and exuberant growth of the mucosa during OM, including the formation of mucosal polyps. Mucosal recovery during OM was also delayed, in parallel with a delay in mucosal apoptosis and reduced caspase gene expression.

**Conclusions:**

The TNF and TNF receptor superfamilies mediate both inflammation and apoptosis during OM. TNF appears to be critical for the maintenance of mucosal architecture in both the normal and infected ME, since excessive accumulation of mucosal tissue is seen in TNFA^-/- ^MEs both before and after bacterial inoculation of the ME. TNFA is also required for appropriate regulation of caspase genes.

## Background

Tumor necrosis factor (TNFA, TNFSF2), formally known as TNFα, is a pleiotropic cytokine widely involved in apoptosis as well as cell proliferation, immune and inflammatory reactions. It is produced by activated macrophages and mast cells, and also by epithelial and stromal cells. TNFA is the founding member of the TNF superfamily, now composed of more than 20 members. Through interaction with their large family of cognate TNF receptors (TNFRs), TNFs can activate transcription factors such as NF-κB and c-Jun, which modulate expression of genes related to apoptosis and various other cellular responses, or via TNFR death domains which can directly stimulate cell death [1-5].

Together with interleukin-1β, TNFA is considered one of the primary cytokines of middle ear (ME) inflammation [6]. In the early stage of inflammation, TNFA is produced by the ME mucosa and in the late stage also by accumulating inflammatory cells. TNFA is induced by bacterial pathogens, both Gram-positive and Gram-negative and it participates in viral otitis media (OM) [7-9]. Elevated levels of TNFA in the ME fluids of patients with OM are very common [10]. In rat and mouse models of acute OM, the expression of TNFA transcripts in the ME mucosa increased substantially within six hours after challenge with nontypeable *Haemophilus influenzae *(NTHi) [11,12]. However, the expression of most other TNF family members and of TNF receptors has not been extensively documented in OM.

TNFA has been demonstrated to up-regulate mucin genes in the ME epithelium [13] and thus probably plays an essential role in the pathogenesis of mucoid OM. Transtympanic injection of TNFA into normal MEs causes OM [14], which can be attenuated by simultaneous administration of TNF soluble receptor type I (TNFsolRI) [15]. Anti-TNFA antibodies have been suggested as a treatment option for OM (reviewed by Smirnova et al. [6]). However, we have previously shown that mice lacking TNFA show a failure to clear bacteria from the ME, accompanied by abnormalities in phagocytosis and intracellular killing by macrophages, and delayed OM resolution [12]. All these findings indicate a broad involvement of TNFA in the inflammatory reaction during OM.

TNFA also plays a major role in apoptosis. Given the delayed resolution of OM observed in TNFA-deficient mice [12], this raises the question of whether TNFA's apoptotic function might contribute to remodeling of the ME mucosa as well as its return to normal structure during recovery from OM. Supporting this concept, dysregulation of TNFA signaling has been reported to be involved in the pathogenesis of nasal polyposis [16] as well as colon polyps [17], reflecting the apoptotic effects of TNFA and alterations of the epithelial architecture due to the accumulation of excess mucosal cells in its absence. Several other members of the TNF and TNFR families are also involved in apoptosis [4,5]. Very few studies have addressed the role of apoptosis, including TNF-mediated cell death, in OM, although it has been demonstrated that mice lacking TNFR6, also known as Fas, show delayed recovery from bacterial OM [18].

To explore the role of the TNF superfamily in regulating apoptosis during OM, we used gene arrays to evaluate the expression of genes encoding all members of the TNF and TNFR superfamilies during NTHi-induced OM in the mouse. In addition, we induced bacterial OM [19] in TNFA^-/- ^and wild-type (WT) mice. We determined the effects of TNFA deletion on mucosal hyperplasia and remodeling as well as upon the course of apoptosis within the ME mucosa during the course of a ME infection.

## Results

### Expression of TNF and TNFR genes

**Figure 1:**
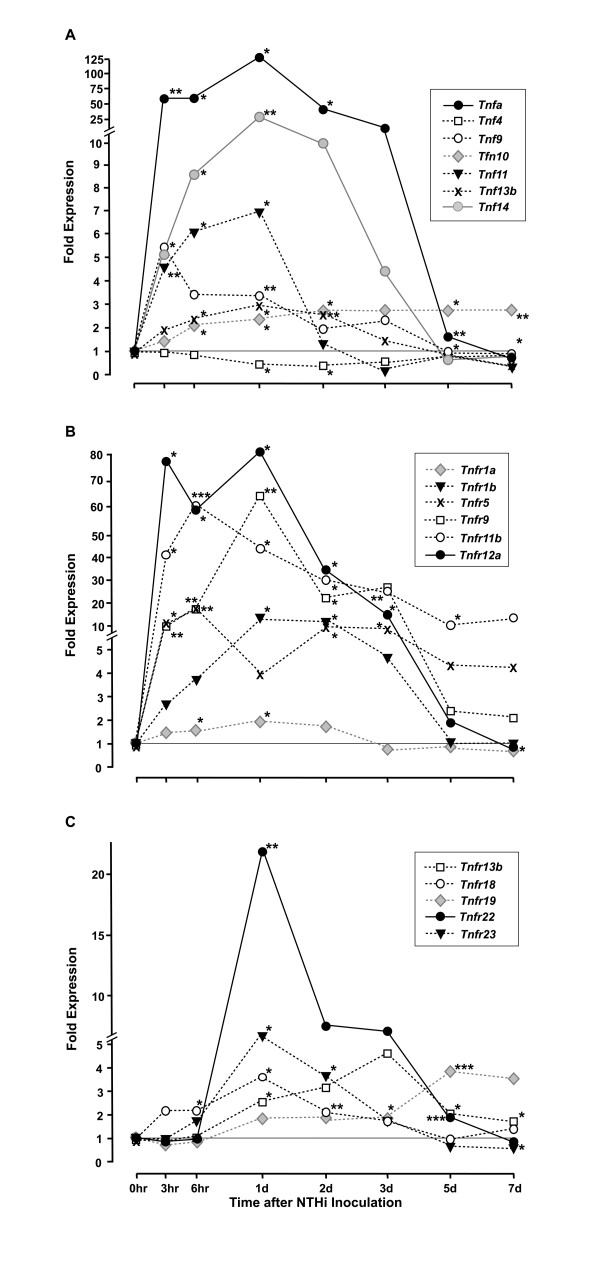
**Relative expression of TNF and TNF receptor superfamily genes during acute OM in the mouse**. **A**. mRNA encoding genes of the 23-member TNF superfamily in the ME mucosa was evaluated on Affymetrix whole-genome gene arrays. Data are expressed as fold induction over untreated ME mucosa (0 h). Only significantly regulated genes are shown. Each data point represents gene arrays obtained from 2 independent sets of 20 C57BL/6J mice each. **B,C**. Relative expression of TNF receptor superfamily genes during acute OM in the mouse. Significantly regulated members of this large (29 member) gene family are represented in the two panels of this figure. = P < .05; ** = P < .01; *** = P < .001.

Of the 23 known TNF superfamily genes, 7 were significantly regulated during OM, as illustrated in Figure 1A, and in the Additional File 1, Table S1. The *Tnfa *gene itself was strongly and significantly up-regulated from 25-fold to more than 100-fold over the period from 3 hours to 3 days after NTHi inoculation, and 2-fold at day 5, when compared to uninoculated (0 hr) controls. *Tnf14 *was up-regulated up to 25-fold, the *Tnf9 *gene over 5-fold, and *Tnf13b *up to 3-fold, over a similar time course. *Tnf11 *was more briefly up-regulated more than 6-fold from 3-24 hours, while *Tnf10 *was up-regulated more than 2-fold at 6 hours and, in contrast to the other genes, remained so throughout the experiment. Only one gene, *Tnf4*, was down-regulated with expression decreased to ~30% of that seen in the control ME 1 and 2 days after NTHi inoculation. Thus, all three TNF family genes with known pro-apoptotic functions (*Tnfa*, *Tnf9 *and *Tnf10*) and one TNF gene with anti-apoptotic function (*Tnf11*) were up-regulated during OM.

Eleven members of the TNFR superfamily were regulated during OM, as illustrated in Figures 1B and 1C, and in the Additional File 1, Table S1. While the *Tnfr1a *and *Tnfr18 *genes were significantly up-regulated from 6 hours to 2 days, the *Tnfr1b*, *Tnfr9*, and *Tnfr12a *genes were much more strongly up-regulated from 3 hours to 3 days. While increasing with a similar pattern, *Tnfr5 *and *Tnfr11b *mRNA appeared to remain elevated until 7 days. *Tnfr22 *and *Tnfr23 *mRNAs were not up-regulated until 1 day after inoculation, and then decreased rapidly. *Tnfr13b *and *Tnfr19 *were also up-regulated relatively late, but remained elevated even at 5 and 7 days after ME infection. Down-regulation of TNFR genes was rare, with only *Tnfr1a *and *Tnfr23 *showing significantly reduced expression at 7 days. Thus, of the TNF receptor genes with known pro-apoptotic function, *Tnfr1a*, *Tnfr1b*, and *Tnfr9 *were up-regulated, as was the anti-apoptotic receptor gene *Tnfr11b*.

### Middle Ear Histology

**Figure 2:**
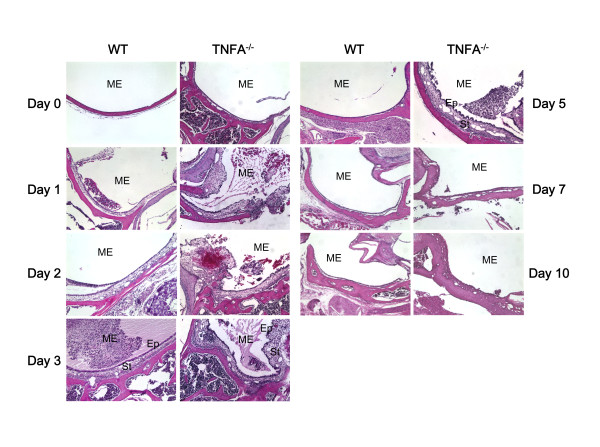
**Middle ear response to NTHi challenge in WT vs. TNF-deficient mice**. H&E stained sections from untreated MEs and MEs at 1, 2, 3, 5, 7 and 10 days after NTHi inoculation. The examples illustrated are representative of the typical response seen in MEs for that time and genotype. However, cellular infiltrates were irregularly distributed so that a match to quantitative data achieved across the entire ME was not always possible. Ep = ME mucosal epithelium. Str = ME mucosal stroma. Original magnification 100×.

**Figure 3:**
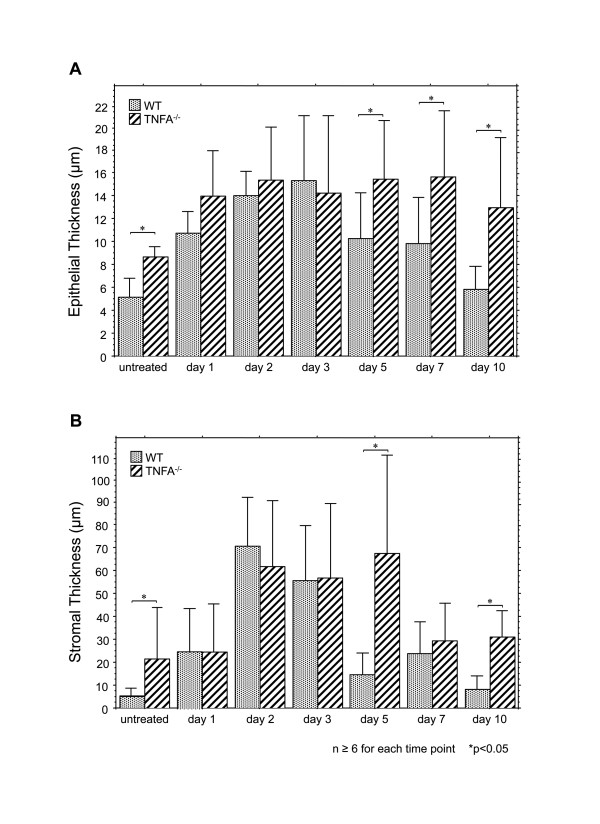
**Thickness of the ME mucosa of WT vs. TNFA**^**-/- **^**mice during NTHi-induced OM**. **A**. Thickness of the ME mucosal epithelium in WT (stippled bars) and TNFA^-/- ^(striped bars) mice. **B**. Thickness of the mucosal stroma. Bars represent means, while lines represent 1 SD. * = p ≤ 0.05; n = 6-10 for each group.

As we have described previously [20-22] in WT mice ME mucosal hyperplasia was observed starting on day 1 and peaked on day 2 after NTHi inoculation. On day 3 the ME mucosa remained significantly hyperplastic, but by day 5 it had recovered to near normal thickness (Figure 2). When the mucosa was divided into its epithelial and stromal compartments however, as shown in Figure 3A and 3B, recovery of epithelial thickness was not complete until day 10, while the stroma had recovered by day 5. The proportion of the ME mucosa occupied by infiltrating leukocytes was highest on day 1, and decreased regularly until few cells were observed on day 5 and virtually none on day 7.

A significant difference between TNFA^-/- ^mice and WT mice was observed even in untreated animals: both epithelial and stromal compartments of the ME mucosa were significantly hyperplastic in the absence of TNFA (Figures 2, 3A,B). Thereafter, the mucosa of both WT and TNFA^-/- ^increased when compared to uninoculated animals (p < .05), such that significant differences between WT and TNFA^-/- ^mice in the thickness of the ME mucosa epithelium or stroma were not observed until day 5 after infection. As noted above, the epithelium of the WT mice began to return to its normal thickness on day 5 and was normal by day 10, while the epithelium of the TNF knockout (KO) mice remained hyperplastic. The ME mucosal stroma of the TNFA^-/- ^animals showed no decrease in thickness on day 5, but did recover somewhat on day 7. However, it remained significantly thicker than the WT even on day 10.

**Figure 4:**
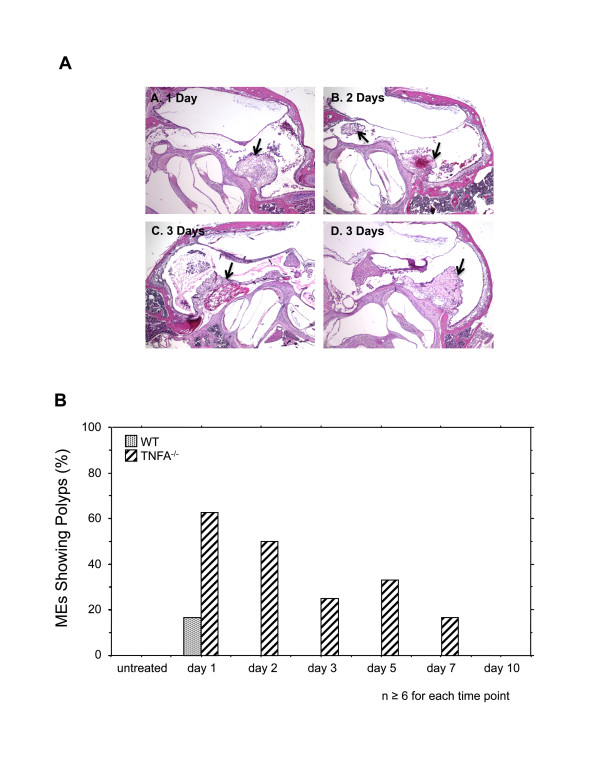
**Mucosal polyps in the mucosa of TNFA**^**-/- **^**mice following NTHi challenge**. **A**. Examples of polyps (arrows) in the ME mucosa of TNF KO mice at various intervals after NTHi inoculation. H&E staining. Magnification A-C = 40x, D = 80x. **B**. Percentage of ears in which polyps were found in serial sections. While polyps were common in TNFA^-/- ^MEs, only one polyp was seen in WT animals. n = 6-10 for each group.

While the thickness of the TNFA^-/- ^ME mucosa in most areas was similar to that in WTs from 1-5 days after inoculation, localized increases in thickness were observed in TNFA^-/- ^mice. Challenge with NTHi resulted in the formation of mucosal polyps in a high percentage of MEs of TNFA^-/- ^mice from 1-7 days after infection. In WT mice, polyp formation was observed in only one ME, on day 1 post infection. Unchallenged animals, either WT or TNFA-deficient, did not exhibit mucosal polyps (Figure 4A and 4B).

**Figure 5:**
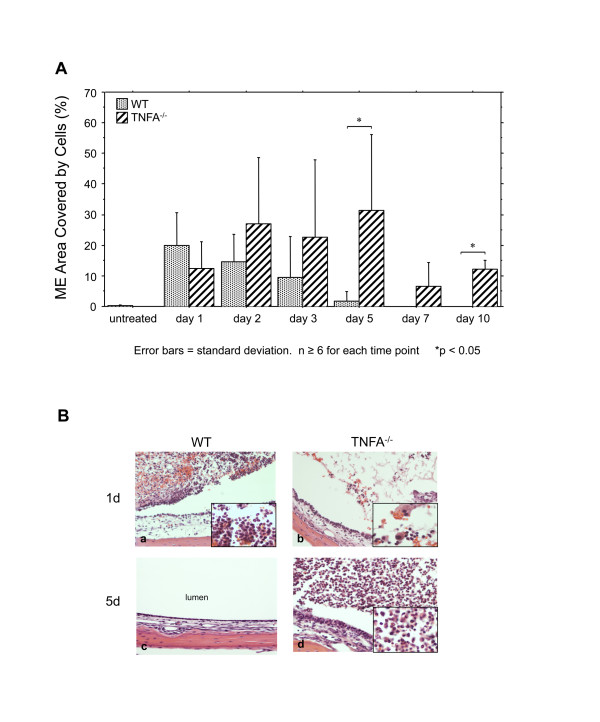
**Leukocyte infiltration of the ME during OM**. **A**. Percentage of the ME lumen occupied by inflammatory cells after challenge with NTHi in WT (stippled bars) and TNFA^-/- ^(striped bars). Bars represent means, while lines represent 1 SD. * = p ≤ 0.05; n = 6-10 for each group. **B**. Micrographs representing the typical appearance of cellular infiltrates in the two genotypes at 1 and 5 days after NTHi inoculation. Magnification 150× (insert = 400×)

Lack of TNF also influenced infiltration of the ME by leukocytes. In TNFA^-/- ^animals, more cells entered the ME. Moreover, apparently viable leukocytes were present in the ME for a longer period (Figure 5A). This difference reached significance by day 5, and remained so on day 10. In addition, the character of the cells was also different (Figure 5B). Almost all of the cells observed in the WT ME on day one were neutrophils, as we have noted previously [12]. In contrast, significant numbers of macrophages were observed in the TNFA^-/- ^MEs, at this time. Moreover, by day 5 when macrophages dominated the few cells remaining in the WT ME, TNFA^-/- ^MEs contained a high proportion of neutrophils.

### Apoptosis

**Figure 6:**
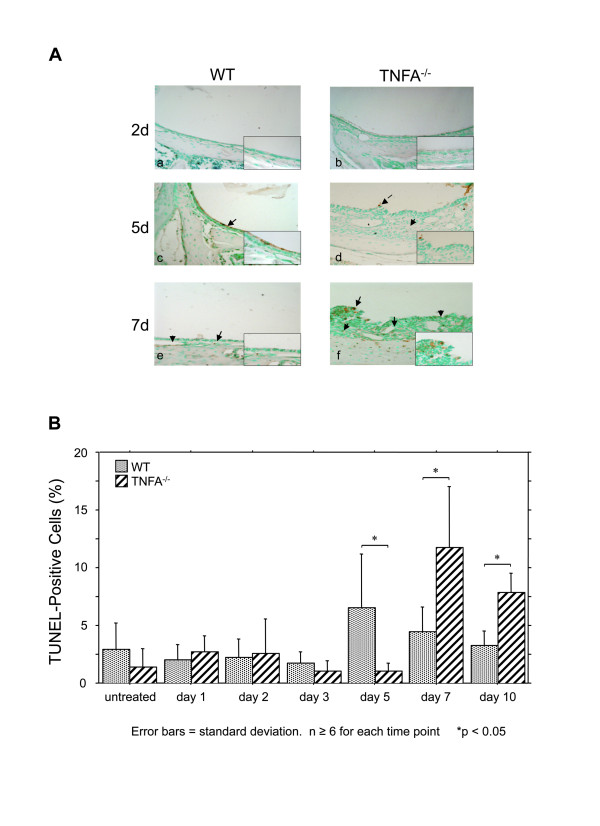
**Apoptosis in the ME mucosa during OM**. **A**. Micrographs illustrating TUNEL-labeled cells in the ME mucosae of WT versus TNFA^-/- ^mice after inoculation of the ME with NTHi. **B**. Percentage of apoptotic cells in the ME mucosa following NTHi injection. Values are means ± SD; asterisks indicate statistical significance (p ≤ 0.05); n = 6-10 for each group.

The time course of apoptosis in the ME mucosa of WT mice, represented by the percentage of TUNEL-positive cells, is illustrated in Figure 6. Apoptotic cells were observed in the mucosal epithelium and stroma (Figure 6A) as well as among infiltrating leukocytes. As expected, counts of apoptotic cells (Figure 6B) revealed a low level of baseline level of apoptosis even in the uninfected WT ME. This was maintained during OM, but showed a single significant peak on day 5 after infection, at the time when mucosal thickening was returning to baseline. In the absence of TNF, increased apoptosis was delayed until 7 days post infection, and remained significantly higher than observed in WTs at 10 days.

**Figure 7:**
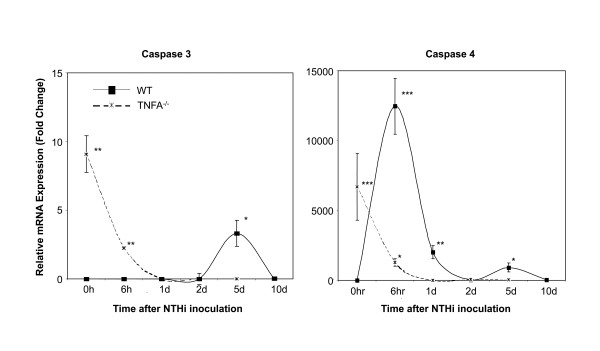
**Expression of caspase mRNAs during NTHi-induced OM**. In WTs, caspase 3 was expressed only on day 5, while caspase 4 mRNA was expressed at high levels early in OM, and then again at day 5. While both caspases were expressed at high levels in TNFA^-/- ^MEs prior to OM, expression declined rapidly and became undetectable soon after inoculation.

To further address the mechanism of changes in apoptosis, we evaluated, using qPCR (Figure 7), the expression of the primary effector caspase, caspase 3, and caspase 4 which serves both inflammatory and apoptotic roles [23,24]. Expression of both caspases was low in uninfected WT MEs. Following ME infection, no change in caspase 3 mRNA was noted until day 5, when expression was up-regulated before returning to its original level by day 10. Caspase 4 showed a strong peak 6 hours after NTHi inoculation in WT mice, followed by a decline and then a second, albeit lower peak at day 5. In TNFA^-/- ^mice, both caspases 3 and 4 were expressed at moderate levels prior to NTHi inoculation. However, after inoculation their expression rapidly decreased to very low levels, and remained so through day 10.

## Discussion

The regulation of multiple TNF and TNFR superfamily genes during OM indicates the importance of these gene families to the disease process and also presumably reflects the pleiotropic nature of TNF/TNFR function. While changes in TNF/TNFR signalling could be mediated by the observed regulation of TNF/TNFR genes, signalling is also likely to be modulated independent of gene regulation through post-translational modification. It is known that post-translational changes in mRNA splicing or in the effector proteins can strongly influence TNF/TNFR interactions [4,5], and we acknowledge that our gene array data do not address this point. However, conclusions can be drawn based on the particular TNFs and TNFRs whose genes did exhibit regulation during OM.

Of the TNF superfamily members encoded by genes that were significantly regulated, TNFA and TNF10 (TRAIL) activate TNF receptors (TNFR1a, TNFR1b, TNFR10) that contain death domains [5,25,26], and thus are likely to induce apoptosis. Of the regulated TNFs that activate receptors without a death domain, TNF9 can enhance apoptosis indirectly by inducing prolonged expression of TNF [27,28]; TNF11 and TNF14 can be anti-apoptotic [29-31], while others appear to have only non-apoptotic functions (TNF4; T-cell recruitment, proliferation and cytokine production [32]; TNF13B, B-cell activation [33]).

Not all of the genes encoding receptors for the above TNFs were themselves regulated, and conversely some receptor genes were regulated in the absence of gene regulation of their ligand. These included TNFR18, which regulates T-cell mediated cell death [34,35], and TNFR19, which can induce apoptosis via JNK signaling [36]. Others encoded decoy receptors that are anti-apoptotic, such as TNFR11B, a secreted receptor that can bind TNF10 [37], and membrane-inserted TNFR22 and TNFR23 [38]. Still others encoded receptors with functions unrelated to apoptosis, including TNFR5 (B-cell growth, differentiation and survival [39]) and TNFR12a (angiogenesis [40]).

It might be hypothesized that apoptosis would be inhibited early in OM to support mucosal hyperplasia, and enhanced during OM recovery to remove excess mucosal cells and leukocytes. Indeed, apoptosis as measured by TUNEL labeling peaked in WT mice late in OM. However, apoptosis was also observed early in OM, and indeed in the normal, uninfected ME. Evaluating the time courses of gene expression, the various pro-apoptotic TNF and TNFR genes that are regulated during acute OM were expressed over its entire time course, beginning at 3 hours and continuing until 7 days. Thus the genes for *Tnfa *and its receptors *Tnfr1a *and *Tnfr1b*, *Tnf9 *and *Tnfr9*, as well as *Tnf1r18 *are up-regulated early in OM and decline after day 3, although *Tnfa *remained 1.8X up-regulated at day 5. *Tnf10 *expression is enhanced throughout OM and that of *Tnfr19 *late in OM. Conversely, the various anti-apoptotic genes are also expressed throughout OM. While *Tnf11 *and *Tnf14 *are expressed early in OM, *Tnfr22 *and *Tnfr23 *are expressed midway through OM, while *Tnfr11b *is up-regulated throughout OM. Of course, the TNFs and their receptors are multifunctional, and no direct relationship between gene expression and apoptosis can be drawn. However, the substrates for both apoptosis enhancement and inhibition were present throughout OM.

During development, apoptosis occurs extensively during tissue remodeling (e.g. [41]), thus it is not surprising that pro-apoptotic genes and apoptosis were observed early in OM as the ME mucosa remodels to adopt a more respiratory phenotype (e.g. [42]). Also, the expression of pro- and anti-apoptotic genes throughout OM is consistent with current concepts of programmed cell death, which must be kept under tight regulation to prevent unwanted loss of cells [e.g. [43,44]]. The peak of TUNEL labeling observed at 5-10 days after NTHi inoculation in WT mice presumably reflects an increase in factors favoring apoptosis and a decrease in anti-apoptotic factors. To the extent that TNFs and TNFRs are responsible, pro-apoptotic TNFA and TNF10 (TRAIL) and TNF19 are good candidates to enhance apoptosis late in OM, while TNFR11B could negatively regulate apoptosis during OM recovery. The increase in TUNEL labeling in the ME mucosa coincides with late changes in the expression of caspase 3 and caspase 4 as observed in the qPCR data from the WT ME on day 5 after inoculation.

Absence of the TNFA gene during bacterial OM had profound effects upon the architecture of the ME mucosa, infiltration of the ME by leukocytes, and apoptosis. Even prior to infection, the ME mucosa was hyperplastic in TNF KO mice, perhaps reflecting reduced apoptosis in the resting ME resulting in the accumulation of excess tissue due to altered homeostatic remodeling. However, while the amount of apoptosis observed in the TNF KO ME mucosa was lower than that seen in the WT ME (Figure 6B), this difference was not statistically significant. Alternatively, hyperplasia in the TNF KO ME could reflect low-grade infection with endogenous microorganisms in the absence of this critical cytokine. We have previously shown that TNFA is critical to bacterial clearance in the ME, and that macrophages of TNFA^-/- ^mice display impaired phagocytosis and intracellular killing of NTHi. In fact, we cultured bacteria from the MEs of naïve TNFA^-/- ^mice on chocolate agar (data not shown). However, we could not identify the organisms and can only speculate regarding their role.

While the ME mucosa of uninfected TNFA^-/- ^mice was significantly thicker than that of WTs, NTHi inoculation nevertheless resulted in significant additional mucosal growth in TNFA^-/- ^animals when compared to the pre-inoculation state. Hyperplasia of the majority of the TNFA^-/- ^ME mucosa that occurred after NTHi inoculation was not significantly different from that seen in WTs for the first few days, suggesting that TNFA does not play a role in initial hyperplasia. An exception was the development of mucosal polyps, which was observed as early as 1 day after inoculation. However, recovery of the mucosa to normal thickness was significantly delayed by lack of TNFA (Figures 2 and 3). The reduced apoptosis observed in the ME mucosa of TNFA^-/- ^mice on day 5, when compared to WTs, seems likely to contribute to both the persistence of mucosal thickness and the development of mucosal polyps (Figure 4). While at later times TNFA^-/- ^animals showed higher levels of apoptosis, this may reflect the fact that recovery is complete in the WT ME, while delayed recovery from hyperplasia continues in TNFA^-/- ^mice. This finding suggests that impaired recovery from ME inflammation in TNFA^-/- ^mice, as found by Leichtle et al. [12] may be the primary reason for persistent ME mucosal thickness in TNFA^-/- ^mice. The lack of caspase 3 and 4 up-regulation on day 5 in TNFA^-/- ^mice, when compared to WTs, also seems likely to reflect this general prolongation of hyperplasia and inflammation. The observation of increased apoptosis on days 7 and 10 (Figure 6B) further suggests that alternative mechanisms for production of mucosal apoptosis must be active in the MEs of TNFA^-/- ^mice, perhaps mediated by other pro-apoptotic members of the TNF superfamily such as TNF10 or, given the results of Rikvin et al. [18], TNF6 (Fas). This apoptosis appears directed at recovery of the ME mucosa, since the delayed peak in apoptosis in TNFA^-/- ^mice 7 days post infection correlates with the time of regression of mucosal polyps (Figure 4).

The prolonged presence of leukocytes in the MEs of TNFA^-/- ^mice is very unlikely to involve lack of TNFA-mediated apoptosis, as TNFA does not appear to mediate cell death in leukocytes. Neutrophils die by an intrinsic program of apoptosis, approximately three days after differentiation, that does not require death ligands [45], while apoptosis of other classes of leukocytes involve alternative TNFs such as TNF6 (Fas). Prolonged inflammation leading to enhanced leukocyte recruitment or lower levels of leukocyte clearance via vessels seem more likely, since TNFA has been shown to be involved in leukocyte trafficking in other systems [46].

Of course, our results are specific to NTHi-induced OM. However, given the similarity or OM induced by NTHi and pneumococcus in the mouse [19], and the central role of TNFA in both inflammation and apoptosis, similar effects of TNFA deletion might be observed in OM induced by *pneumococcus *and perhaps other ME pathogens.

## Conclusions

The results of the present study suggest that the TNF and TNF receptor superfamilies play important roles in OM, mediating both inflammation and apoptosis. TNFA appears to be a critical factor for the maintenance of mucosal architecture in both the normal and infected ME since excessive accumulation of mucosal tissue is seen in TNFA^-/- ^MEs both before and after bacterial inoculation of the ME.

## Methods

### Animals

6 week old female TNFA^-/- ^mice (B6;129S6-^Tnftm1Gkl^/J) were obtained from Jackson Labs (Bar Harbor, ME) and housed under germ-free conditions. These mice are homozygous for a targeted mutation in the TNF gene (*Tnfa, Tnfsf2*, *Tnftm1Gkl*) and are therefore TNFA deficient [20,47]. Female B6;129SF2 mice served as WT controls. These mice provide a close match for the B6;129 background of the TNFA^-/- ^mice. All animal experimental procedures followed the appropriate NIH guidelines. They were reviewed and approved by the Institutional Animal Use and Care Committee of the San Diego VA Medical Center, protocol number 07-024.

### Bacteria

*Haemophilus influenzae *strain 3655 (nontypeable, biotype II; NTHi) was used at a concentration of 10^5 ^- 10^6^/ml to induce experimental OM. The inocula were prepared as previously described [19,21].

### Surgery

Anesthesia was induced by intraperitoneal injection of 0.4 ml per 100 g bodyweight of a rodent cocktail containing 13.3 mg/ml ketamine HCl, 1.3 mg/ml xylazine and 0.25 mg/ml acepromazine maleat. The ME bullae were exposed bilaterally through a vertical midline incision on the neck. A 25 gauge syringe needle was used to fenestrate the center of the ME bullae. Via this opening, approximately 3-5 μl of NTHi (10^5^/ml) was injected using an insulin syringe. Excess fluid was absorbed with a sterile cotton swab, the cervical tissue was replaced over the fenestrations and the skin incision stapled closed. The mice were allowed to survive for various intervals after inoculation.

### Gene Expression

Expression of TNF and TNFR family genes was evaluated in WT mice by DNA microarray. Age-matched C57Bl/6:CB F1 hybrid mice were purchased from Jackson Laboratories (Bar Harbor, ME). Twenty mice per time point were inoculated bilaterally with *Haemophilus influenzae *strain 3655 (non-typeable, biotype II, originally isolated from an OM patient; NTHi), in 5 ml at a concentration of 10^5 ^- 10^6^/ml as described previously [19,21]. Uninoculated, naive animals (time 0) served as controls. ME mucosae were harvested and combined at each of the following intervals: 0 (no treatment), 3 or 6 hours, as well as 1, 2, 3, 5 or 7 days after NTHi inoculation. The tissue was homogenized in TRIzol™ (Invitrogen, Carlsbad, CA) and total RNA was extracted. Total RNA quality was assessed using the RNA 6000 Labchip Kit on the Agilent 2100 Bioanalyzer for the integrity of 18S and 28S ribosomal RNA. The mRNA was reverse transcribed using a T7-oligodT primer then in vitro transcribed using T7 RNA polymerase to generate biotinylated cRNA probes that were hybridized to two Affymetrix MU430 2.0 microarrays per sample. This procedure was then duplicated for each time point to obtain a second, independent replication, for a total of 32 arrays for the controls and seven OM time points.

Expression of mRNA encoding caspases 3 and 4 was evaluated in WT and TNFA^-/- ^mice using qPCR. Mice were inoculated with NTHi and sacrificed at intervals of 0 or 6 hours, as well as 1, 2, 5 or 10 days. The contents of at least 6 MEs per time point and genotype were isolated. mRNA was extracted, reverse transcribed, and 1 mg/ml of cDNA was amplified using pre-developed TaqMan qPCR primers (Applied Biosystems, Foster City, CA) for caspase 3 (Mm01195084_m1) and caspase 4 (Mm00432307_m1) in an ABI Prism 7000 Sequence Detection System (Applied Biosystems). Fold induction was calculated using the comparative threshold cycle (Ct) method [48]. Relative expression of each target gene was normalized to levels of GAPDH (Mm03302249_g1) and compared to uninfected mucosa.

### Histology

The mice were sacrificed by intracardial perfusion with PBS and 4% paraformaldehyde (PFA) under general anesthesia, 1, 2, 3, 5, 7 or 10 days after inoculation. The MEs were isolated and postfixed in 4% PFA overnight. The ME bullae were decalcified for 14 days in EDTA and embedded in paraffin. Serial 7 μm sections were stained with H&E. Digital micrographs were taken from a standardized region of the ME of each specimen. The thickness of the epithelial and stromal layers of the mucosa was measured over approximately 500 μm at a standard location in the plane of the Eustachian tube and computer-averaged.

For analysis of the number of inflammatory cells, digital micrographs were obtained from each specimen in the plane that displayed the maximum amount of cells in the ME cavity. The area covered by inflammatory cells as a percentage of a standard area in the ME cavity was then calculated using image analysis software.

### Apoptosis

For TUNEL labeling, paraffin sections of each ear were deparaffinized and labeled with the "TACS™ 2 TdT-DAB In Situ Apoptosis Detection Kit" (Trevigen, Inc., Gaithersburg, MD) including positive and negative controls. The percentage of TUNEL positive cells was determined by enumerating 100 mucosal cells in three different planes in each specimen. Each ear was also examined for polyp formation in the ME mucosa in H&E stained serial sections, as a measure of the accumulation of excess mucosal cells. The percentage of ears with polyps was calculated. All calculations were performed independently, by two observers, with matching results.

### Statistics

For statistical evaluation of gene array data, raw intensity data was median normalized and statistical differences in gene transcript expression levels were evaluated using a variance-modeled posterior inference approach (VAMPIRE) [49]. Specific TNF and TNFR family genes were assessed at individual time points, after Bonferonni correction for multiple tests, using Genespring GX 7.3 (Agilent). To quantify differential gene expression, transcript hybridization levels were expressed as fold change relative to those obtained for uninfected control (0 hr) MEs. In the text they have been described as up-regulated if they were significantly higher than control values, and down-regulated if they were significantly lower. To document statistical significance of morphological and cellular data, ANOVA was performed on all data using StatView 5.0 statistics calculation software. Differences between groups were considered significant at p < 0.05.

## Abbreviations

Ct: comparative threshold cycle; ME: middle ear; KO: knockout; NTHi: nontypeable *Haemophilus influenzae*; OM: otitis media; PFA: paraformaldehyde; TNFA: tumor necrosis factor α; TNFA^-/- ^mice: TNF-deficient (B6129S6-^Tnftm1Gkl^/J) mice; TNFR: TNF receptor; TNFsolRI: TNF soluble receptor type I; VAMPIRE: variance-modeled posterior inference; WT: wild-type mice, B6129SF2.

## Authors' contributions

JE and AL contributed to experimental design, performed the majority of the experiments, analyzed data and wrote the manuscript. MH and KP assisted with knockout mouse and gene array experiments and data analysis. UE participated in animal surgery and preparation of histologic specimens. JH performed gene array data analysis. DB provided advice on experimental design. HS assisted in histology, participated in composing the manuscript and gave advice in data interpretation. AFR and SIW designed the study, provided overall supervision, assisted with data analysis and interpretation, and made substantive contributions to writing the manuscript. All authors read and approved the final manuscript.

## Supplementary Material

Additional file 1 **Table S1**. Fold expression data derived from gene arrays, for the TNF and TNFR family genes presented graphically in Figure 1, including variability (Range) and significance (P-value). Click here for file
