# Supplementary material for: TNFA deletion alters apoptosis as well as caspase 3 and 4 expression during otitis media
Source: BMC Immunol. 2011 Jan 26;12:12. doi: 10.1186/1471-2172-12-12 (PMC3040143; doi:10.1186/1471-2172-12-12)
Supplement: Additional file 1 — Table S1. Fold expression data derived from gene arrays, for the TNF and TNFR family genes presented graphically in Figure 1, including variability (Range) and significance (P-value). [file 1471-2172-12-12-S1.DOC]

| **TABLE S1: TNF - TNFR Gene Expression During Otitis Media (OM)** | | | | | | | | | |
| --- | --- | --- | --- | --- | --- | --- | --- | --- | --- |
|  |  |  |  |  |  |  |  |  |  |
| **1. TNF Genes Regulated During OM** | | | | |  |  |  |  |  |
|  | Time: | 0h | 3h | 6h | 24h | 2d | 3d | 5d | 7d |
| *Tnfa* (1419607_at) | | |  |  |  |  |  |  |  |
|  | Fold Exp. | 1.0 | 63.8 | 63.4 | 130.9 | 38.0 | 20.8 | 1.8 | 0.9 |
|  | Range | 0.7-1.3 | 61.9-65.8 | 49.6-81.2 | 120.8-141.9 | 35.8-40.3 | 14.1-30.7 | 1.5-2.2 | 0.6-1.1 |
|  | P-Value | 0.91 | **0.00** | **0.04** | **0.01** | **0.01** | 0.08 | 0.21 | 0.69 |
|  |  |  |  |  |  |  |  |  |  |
| *Tnf4* (1421744_at) | | |  |  |  |  |  |  |  |
|  | Fold Exp. | 1.0 | 1.0 | 0.8 | 0.4 | 0.4 | 0.6 | 0.7 | 0.8 |
|  | Range | 0.9-1.1 | 1.0-1.0 | 0.7-1.0 | 0.4-0.5 | 0.9-0.4 | 0.5-0.6 | 0.7-0.8 | 0.7-0.9 |
|  | P-Value | 0.97 | 0.13 | 0.45 | **0.03** | **0.02** | 0.13 | 0.11 | 0.33 |
|  |  |  |  |  |  |  |  |  |  |
| *Tnf9* (1422924_at) | | |  |  |  |  |  |  |  |
|  | Fold Exp. | 0.9 | 5.4 | 3.3 | 3.0 | 1.9 | 2.5 | 1.0 | 0.9 |
|  | Range | 0.7-1.4 | 5.2-5.7 | 3.0-3.7 | 3.0-3.0 | 1.7-2.2 | 2.4-2.5 | 1.0-1.0 | 0.9-1.0 |
|  | P-Value | 0.89 | **0.02** | 0.05 | **0.01** | 0.14 | **0.01** | 0.87 | 0.62 |
|  |  |  |  |  |  |  |  |  |  |
| *Tnf10* (1420412_at) | | |  |  |  |  |  |  |  |
|  | Fold Exp. | 1.0 | 1.5 | 2.2 | 2.4 | 2.7 | 2.6 | 2.6 | 2.6 |
|  | Range | 0.8-1.2 | 1.2-1.8 | 2.1-2.3 | 2.2-2.5 | 2.5-2.8 | 2.3-3.0 | 2.5-2.8 | 2.0-3.4 |
|  | P-Value | 0.94 | 0.29 | **0.04** | **0.04** | **0.03** | 0.08 | **0.03** | 0.18 |
|  |  |  |  |  |  |  |  |  |  |
| *Tnf11* (1451944_a_at) | | |  |  |  |  |  |  |  |
|  | Fold Exp. | 1.0 | 4.6 | 6.0 | 6.8 | 1.2 | 0.3 | 0.7 | 0.4 |
|  | Range | 0.8-1.2 | 4.5-4.7 | 5.7-6.3 | 5.9-7.8 | 0.8-1.9 | 0.1-0.7 | 0.5-1.1 | 0.09-2.1 |
|  | P-Value | 0.93 | **0.01** | **0.01** | 0.05 | 0.73 | 0.42 | 0.60 | 0.69 |
|  |  |  |  |  |  |  |  |  |  |
| *Tnf13b* (1460255_at) | | |  |  |  |  |  |  |  |
|  | Fold Exp. | 1.0 | 1.6 | 2.4 | 3.0 | 2.2 | 1.3 | 0.9 | 0.6 |
|  | Range | 0.8-1.2 | 1.5-1.8 | 2.2-2.5 | 2.9-3.1 | 2.2-2.2 | 1.1-1.5 | 0.8-1.0 | 0.5-0.7 |
|  | P-Value | 0.95 | 0.12 | **0.04** | **0.01** | **0.01** | 0.33 | 0.46 | 0.22 |
|  |  |  |  |  |  |  |  |  |  |
| *Tnf14* (1450298_at) | | |  |  |  |  |  |  |  |
|  | Fold Exp. | 0.9 | 5.2 | 8.6 | 31.1 | 9.9 | 3.8 | 0.7 | 0.7 |
|  | Range | 0.6-1.4 | 2.8-9.4 | 8.3-9. | 29.5-32.8 | 7.9-12.4 | 3.3-4.5 | 0.3-1.9 | 0.6-0.7 |
|  | P-Value | 0.86 | 0.22 | **0.01** | **0.01** | 0.06 | 0.07 | 0.80 | **0.04** |
|  |  |  |  |  |  |  |  |  |  |
| **2. TNF Receptor Genes Regulated During OM** | | | | | |  |  |  |  |
|  | Time: | 0h | 3h | 6h | 24h | 2d | 3d | 5d | 7d |
| *Tnfr1a* (1417281_at) | | |  |  |  |  |  |  |  |
|  | Fold Exp. | 1.0 | 1.5 | 1.6 | 1.9 | 1.6 | 0.8 | 0.9 | 0.7 |
|  | Range | 0.9-1.1 | 1.4-1.5 | 1.6-1.6 | 1.8-2.0 | 1.3-1.8 | 0.7-0.8 | 0.9-1.0 | 0.7-0.7 |
|  | P-Value | 0.97 | 0.06 | **0.02** | **0.04** | 0.21 | 0.17 | 0.22 | **0.04** |
|  |  |  |  |  |  |  |  |  |  |
| *Tnfr1b* (1418099_at) | | |  |  |  |  |  |  |  |
|  | Fold Exp. | 1.0 | 2.7 | 3.7 | 12.5 | 5.8 | 4.4 | 1.1 | 1.1 |
|  | Range | 1.0-1.0 | 2.3-3.1 | 3.6-3.8 | 12.3-12.8 | 4.5-7.4 | 3.6-5.3 | 1.1-1.2 | 1.1-1.1 |
|  | P-Value | 0.99 | 0.09 | **0.01** | **0.00** | 0.09 | 0.09 | 0.18 | 0.22 |
|  |  |  |  |  |  |  |  |  |  |
| **2. TNF Receptor Genes Regulated during OM (continued)** | | | | | | |  |  |  |
|  | Time: | 0h | 3h | 6h | 24h | 2d | 3d | 5d | 7d |
| *Tnfr5* (1439921_s_at) | | |  |  |  |  |  |  |  |
|  | Fold Exp. | 0.9 | 12.1 | 18.3 | 3.6 | 11.9 | 12.0 | 4.3 | 4.2 |
|  | Range | 0.6-1.4 | 10.4-14.1 | 18.1-18.4 | 2.9-4.6 | 10.8-13.2 | 10.9-13.1 | 3.3-5.7 | 3.8-4.6 |
|  | P-Value | 0.89 | **0.04** | **0.00** | 0.12 | **0.03** | **0.02** | 0.12 | **0.05** |
|  |  |  |  |  |  |  |  |  |  |
| *Tnfr9* (1428034_a_at) | | |  |  |  |  |  |  |  |
|  | Fold Exp. | 0.8 | 10.5 | 18.9 | 64.4 | 22.1 | 27.9 | 2.6 | 2.3 |
|  | Range | 0.4-1.6 | 10.2-10.9 | 18.8-19.1 | 61.8-67.1 | 17.4-28.1 | 21.4-36.5 | 1.8-3.6 | 1.8-2.9 |
|  | P-Value | 0.80 | **0.01** | **0.00** | **0.01** | **0.05** | 0.05 | 0.21 | 0.17 |
|  |  |  |  |  |  |  |  |  |  |
| *Tnfr11b* (1418309_at) | | |  |  |  |  |  |  |  |
|  | Fold Exp. | 1.0 | 39.5 | 60.8 | 43.1 | 28.6 | 27.0 | 9.1 | 13.4 |
|  | Range | 0.8-1.2 | 33.4-46.9 | 60.6-61.0 | 38.3-48.5 | 23.2-35.3 | 26.3-27.8 | 8.2-10.0 | 10.4-17.4 |
|  | P-Value | 0.93 | **0.03** | **0.00** | **0.02** | **0.04** | **0.01** | **0.03** | 0.06 |
|  |  |  |  |  |  |  |  |  |  |
| *Tnfr12a* (1418571_at) | | |  |  |  |  |  |  |  |
|  | Fold Exp. | 0.9 | 77.3 | 58.3 | 81.8 | 31.8 | 16.2 | 1.9 | 1.5 |
|  | Range | 0.5-1.5 | 70.6-84.5 | 46.2-73.6 | 73.2-91.5 | 29.9-33.9 | 13.5-19.5 | 1.7-2.2 | 1.0-2.1 |
|  | P-Value | 0.84 | **0.01** | **0.04** | **0.02** | **0.01** | **0.04** | 0.13 | 0.49 |
|  |  |  |  |  |  |  |  |  |  |
| *Tnfr13b* (1423182_at) | | |  |  |  |  |  |  |  |
|  | Fold Exp. | 1.0 | 0.8 | 1.2 | 2.4 | 3.0 | 4.1 | 2.0 | 1.7 |
|  | Range | 1.0-1.0 | 0.7-1.0 | 1.1-1.3 | 2.2-2.5 | 2.2-4.2 | 3.5-4.9 | 2.0-2.1 | 1.6-1.8 |
|  | P-Value | 0.99 | 0.36 | 0.25 | **0.03** | 0.18 | 0.07 | **0.02** | 0.05 |
|  |  |  |  |  |  |  |  |  |  |
| *Tnfr18* (1422303_a_at) | | |  |  |  |  |  |  |  |
|  | Fold Exp. | 1.0 | 2.2 | 2.2 | 3.4 | 2.0 | 1.5 | 0.9 | 1.3 |
|  | Range | 1.0-1.0 | 2.0-2.5 | 2.1-2.3 | 3.3-3.5 | 2.0-2.1 | 1.3-1.7 | 0.9-1.0 | 1.1-1.4 |
|  | P-Value | 0.99 | 0.08 | **0.04** | **0.02** | **0.01** | 0.21 | 0.48 | 0.26 |
|  |  |  |  |  |  |  |  |  |  |
| *Tnfr19* (1415921_a_at) | | |  |  |  |  |  |  |  |
|  | Fold Exp. | 1.0 | 0.8 | 0.8 | 1.7 | 1.9 | 1.9 | 3.3 | 3.1 |
|  | Range | 0.7-1.3 | 0.6-1.1 | 0.7-1.0 | 1.7-1.8 | 1.8-2.0 | 1.8-2.0 | 3.3-3.3 | 2.4-3.9 |
|  | P-Value | 0.91 | 0.57 | 0.57 | **0.04** | 0.06 | **0.05** | **0.00** | 0.13 |
|  |  |  |  |  |  |  |  |  |  |
| *Tnfr22* (1422038_a_at) | | |  |  |  |  |  |  |  |
|  | Fold Exp. | 1.0 | 0.9 | 1.2 | 22.2 | 6.1 | 5.8 | 1.9 | 0.9 |
|  | Range | 1.0-1.0 | 0.6-1.2 | 1.0-1.3 | 21.8-22.6 | 3.0-12.2 | 4.5-7.4 | 1.9-1.9 | 0.6-1.4 |
|  | P-Value | 0.99 | 0.76 | 0.41 | **0.00** | 0.24 | 0.09 | **0.00** | 0.87 |
|  |  |  |  |  |  |  |  |  |  |
| *Tnfr23* (1422101_at) | | |  |  |  |  |  |  |  |
|  | Fold Exp. | 1.0 | 1.0 | 1.4 | 5.2 | 3.4 | 1.8 | 0.8 | 0.6 |
|  | Range | 0.8-1.2 | 0.9-1.2 | 1.3-1.5 | 4.8-5.5 | 3.1-3.6 | 1.1-3.0 | 0.6-1.0 | 0.4-0.9 |
|  | P-Value | 0.94 | 0.82 | 0.11 | **0.03** | **0.04** | 0.46 | 0.53 | 0.41 |
|  |  |  |  |  |  |  |  |  |  |
